# Frontotemporal Dementia & the Criminal Legal System: Care Partner Stories

**DOI:** 10.1002/alz70858_098222

**Published:** 2025-12-24

**Authors:** Victoria Helmly

**Affiliations:** ^1^ Georgia State University, Atlanta, GA, USA

## Abstract

**Background:**

Research has established that individuals affected by frontotemporal dementia (FTD) may have symptoms, including behavioral symptoms, which violate social norms and may be considered “criminal.” As a result, persons living with FTD may be more vulnerable and at risk for interactions with the criminal legal system, including arrests and incarceration.

**Method:**

Semi‐structured interviews were conducted with care partners for people with FTD who have had contact with the criminal legal system in the United States. Interviews were transcribed and preliminary thematic coding was performed.

**Result:**

Emergent themes include stress and anxiety about their loved one's interaction with the police, dwindling social support due to their loved one's symptoms, the role of education and advocacy as a protective factor, and the need for more training and awareness about dementia for the criminal legal system.

**Conclusion:**

Preliminary analysis reveals that care partners of individuals with FTD who have had contact with the criminal legal system have diverse experiences and perspectives. Despite this heterogeneity, they face common barriers, mostly due to a lack of awareness and education about the criminal legal system. Care partners rely on shared strengths such as education, social capital, and advocacy skills to navigate these challenges effectively.

